# Supplementary material for: Validation of the Polar V800 heart rate monitor and comparison of artifact correction methods among adults with hypertension
Source: PLoS One. 2020 Oct 8;15(10):e0240220. doi: 10.1371/journal.pone.0240220 (PMC7544136; doi:10.1371/journal.pone.0240220)
Supplement: S3 Dataset — (PDF) [file pone.0240220.s003.pdf]

Raw HRV measures calculated from ECG R-R Intervals (mean±SD).

Subjects	SDNN (ms)	RMSSD (ms)	pNN50 (%)	LFpow_ (ms2)	HFpow_ (ms2)	LFpow_(n.u.)	HFpow_(n.u.)	LF_HF_ratio	SampEn
ECG_1	92.370	70.28868	44.485294	1926.086622	1806.926985	51.575393	48.38462	1.065946	1.614825
ECG_2	66.492	54.35423	24.311927	1793.760301	592.277006	75.095941	24.795732	3.028583	1.483388
ECG_3	86.830	90.44291	43.40836	2225.026267	2866.633358	43.673518	56.267185	0.776181	1.706844
ECG_4	61.121	27.84677	6.970509	2809.761759	142.87056	95.151068	4.838235	19.666485	1.151632
ECG_5	83.655	46.10577	20.833333	1698.185164	531.080103	76.150846	23.814953	3.197606	1.608887
ECG_6	26.762	13.29215	0*	196.549673	82.129733	70.506006	29.461455	2.393161	1.719913
ECG_7	82.467	22.51605	1.759531	971.441618	116.155152	89.291173	10.676534	8.363311	1.244299
ECG_8	19.598	11.13726	0	130.659583	76.175329	63.156772	36.820781	1.715248	1.614524
ECG_9	30.104	16.44595	0.724638	376.501869	92.232597	80.310667	19.673904	4.082091	1.568277
ECG_10	128.186	126.7646	59.530792	6483.635391	5929.982674	52.204518	47.746653	1.093365	1.58529
ECG_11	66.795	37.65209	16.531165	1550.680574	353.587208	81.357042	18.551086	4.385568	1.696386
ECG_12	55.090	48.48735	12.010444	1043.580134	787.503516	56.905012	42.9415	1.325175	1.530052
ECG_13	64.631	90.47792	26.984127	763.035804	1178.395968	39.172765	60.496543	0.647521	0.95915
ECG_14	32.598	25.23615	3.75	162.798857	316.392823	33.945264	65.971211	0.514547	1.649283
ECG_15	43.710	20.83467	1.502146	1008.728056	140.366233	87.724195	12.206972	7.186401	1.144364
ECG_16	23.351	8.069013	0	127.955575	35.815678	78.11207	21.864126	3.572613	1.316848
ECG_17	73.439	33.57606	11.411411	2262.138126	279.276716	88.99091	10.986548	8.099988	1.46879
ECG_18	49.106	40.21488	14.133333	397.561603	1147.719227	25.70811	74.216654	0.346393	1.590132
ECG_19	67.547	33.9669	11.167513	1330.948251	457.576741	74.357822	25.564037	2.908689	1.553912
ECG_20	19.980	9.320905	0.239234	193.313275	35.946281	84.293729	15.674278	5.377838	1.39126
ECG_21	48.331	33.40162	11.278195	1242.158292	614.648112	66.884451	33.095944	2.020926	1.444812
ECG_22	59.527	47.06744	25.581395	1018.87503	610.557981	62.472342	37.436374	1.66876	1.814808
ECG_23	45.101	22.26671	2.553191	196.890808	181.205253	52.023901	47.879351	1.086562	1.860018
ECG_24	28.446	13.12485	0.253807	215.595495	82.534761	72.27272	27.667608	2.612178	1.504606
ECG_25	32.638	14.56898	0.588235	249.539866	61.785393	80.119477	19.837365	4.038817	1.769031

HRV: Heart Rate Variability; ECG: Electrocardiogram; SDNN: standard deviation of normal-to-normal N-N intervals; RMSSD: root mean square of the successive difference of intervals; pNN50%: the percentage of successive normal cardiac inter-beat intervals greater than 50 ms; LF: low frequency; HF: high frequency; SampleEn: Sample Entropy. *This individual did not have R-R intervals >50 ms.

Raw HRV measures calculated from UN Polar V800^{TM} R-R Intervals (mean±SD).

Subjects	SDNN (ms)	RMSSD (ms)	pNN50 (%)	LFpow_ (ms2)	HFpow_ (ms2)	LFpow_(n.u.)	HFpow_(n.u.)	LF_HF_ratio	SampEn
POLAR_UN_1	92.246347	69.929364	44.485294	1912.906924	1776.386131	51.82974	48.130743	1.076853	1.691331
POLAR_UN_2	66.499212	54.443272	24.367816	1787.755628	592.937916	75.011556	24.878789	3.015081	1.467467
POLAR_UN_3	283.570122	390.829339	42.765273	85261.3937	20557.56565	80.532241	19.417309	4.147446	0.604146
POLAR_UN_4	60.849549	28.044931	7.277628	2865.170071	140.841666	95.303894	4.684804	20.343199	1.1418
POLAR_UN_5	148.529615	131.041398	21.305842	3574.895453	5068.754063	41.334746	58.607494	0.705281	0.791147
POLAR_UN _6	26.645059	13.127331	0	195.66382	78.700969	71.291552	28.675277	2.486168	1.758126
POLAR_UN _7	82.414532	22.3191	1.466276	969.142792	114.719045	89.387382	10.580933	8.447968	1.228302
POLAR_UN _8	19.931588	13.321826	0.561798	130.070156	74.460391	63.55993	36.385728	1.746837	1.516484
POLAR_UN _9	30.05709	16.116018	0.724638	376.78453	87.808525	81.087432	18.897187	4.290979	1.52678
POLAR_UN _10	128.155219	127.265847	60.588235	6484.53294	5965.020987	52.060335	47.889493	1.087093	1.60392
POLAR_UN _11	66.555038	37.785131	16.847826	1548.043753	348.46607	81.553963	18.357872	4.442452	1.75275
POLAR_UN _12	152.224462	204.342776	17.119565	12346.43965	15972.85296	43.514843	56.296083	0.772964	0.685451
POLAR_UN _13	143.885688	202.002783	29.220779	9810.725528	12105.63193	44.705551	55.16299	0.810427	0.484184
POLAR_UN _14	32.679642	25.463591	3.75	162.419078	321.050776	33.565426	66.348154	0.505898	1.657119
POLAR_UN _15	43.653315	20.58099	1.502146	1000.913286	137.548093	87.860494	12.074016	7.276824	1.136733
POLAR_UN _16	23.315234	7.942927	0	123.742084	36.02568	77.432968	22.543465	3.43483	1.323371
POLAR_UN _17	128.143856	137.365661	16.167665	3619.812744	7309.37404	33.078514	66.794404	0.495229	0.79712
POLAR_UN _18	48.404648	39.992628	14.438503	393.511389	1147.630165	25.51428	74.409429	0.34289	1.523997
POLAR_UN _19	68.02752	34.903479	12.011173	1392.939434	456.401632	75.260781	24.659466	3.052004	1.528219
POLAR_UN _20	128.387209	182.498569	5.665025	9004.895699	12617.0803	41.564405	58.23737	0.713707	0.2199
POLAR_UN _21	170.534855	233.074927	16.666667	15207.75085	19243.58745	44.111951	55.818392	0.790276	0.719875
POLAR_UN _22	59.453687	47.023268	26.333333	999.065027	603.386144	62.287172	37.618389	1.655764	1.85645
POLAR_UN _23	45.144881	22.644949	3.404255	198.203904	183.180486	51.918725	47.9834	1.082014	1.763296
POLAR_UN _24	151.567907	205.7175	8.80829	13032.92148	15698.41583	45.246258	54.500026	0.830206	0.240347
POLAR_UN _25	60.64545	52.555788	1.169591	704.060416	886.39403	44.119479	55.545294	0.794297	0.757688

UN: Uncorrected R-R intervals imported from Polar V800^{TM}.

Raw HRV measures calculated from Kubios HRV Premium (ver.3.2) AC Polar V800™ R-R Intervals (mean±SD).

Subjects	SDNN (ms)	RMSSD (ms)	pNN50 (%)	LFpow_ (ms2)	HFpow_ (ms2)	LFpow_(n.u.)	HFpow_(n.u.)	LF_HF_ratio	SampEn
POLAR_AC_1	93.19109	69.99802	44.68865	1912.907	1776.386	51.82974	48.13074	1.076853	1.612398
POLAR_AC_2	66.43713	54.40181	24.31193	1788.846	593.0971	75.01797	24.87243	3.016109	1.459025
POLAR_AC_3	75.75456	67.47272	42.76527	1618.276	1634.618	49.72846	50.23064	0.990002	1.935366
POLAR_AC_4	60.84045	28.10463	7.258065	2987.663	157.8171	94.97181	5.016689	18.93117	1.137186
POLAR_AC_5	105.5875	47.74351	21.99313	1833.695	755.7999	70.77902	29.17322	2.426164	1.223424
POLAR_AC _6	26.64506	13.12733	0	195.6638	78.70097	71.29155	28.67528	2.486168	1.713057
POLAR_AC _7	82.41453	22.3191	1.466276	969.1428	114.719	89.38738	10.58093	8.447968	1.234187
POLAR_AC _8	19.50686	11.01503	0	131.0879	73.53491	64.04819	35.92839	1.782663	1.486195
POLAR_AC _9	30.05709	16.11602	0.724638	376.7845	87.80853	81.08743	18.89719	4.290979	1.577162
POLAR_AC _10	128.1552	127.2658	60.58824	6484.533	5965.021	52.06034	47.88949	1.087093	1.579544
POLAR_AC _11	66.7831	37.749	16.80217	1551.825	350.1889	81.51344	18.39453	4.431394	1.71277
POLAR_AC _12	48.24458	35.10544	11.34565	731.4545	498.485	59.41251	40.48953	1.467355	1.621018
POLAR_AC _13	42.88491	33.92338	7.643312	670.0888	251.3477	72.61184	27.23642	2.665983	1.136837
POLAR_AC _14	32.67964	25.46359	3.75	162.4191	321.0508	33.56543	66.34815	0.505898	1.656603
POLAR_AC _15	43.60694	20.58187	1.498929	1000.913	137.5481	87.86049	12.07402	7.276824	1.175465
POLAR_AC _16	23.33778	7.933189	0	125.7425	35.71065	77.86363	22.11314	3.521147	1.342291
POLAR_AC _17	93.90744	41.43503	13.47305	2666.221	558.1217	82.64809	17.30077	4.777133	1.261563
POLAR_AC _18	49.03554	40.1668	14.66667	395.6216	1144.698	25.66485	74.25911	0.345612	1.670536
POLAR_AC _19	67.5676	34.01589	11.16751	1327.143	458.1968	74.27682	25.6441	2.896449	1.508982
POLAR_AC _20	20.10076	9.198699	0.241546	193.0984	32.23874	85.66763	14.30264	5.98964	1.433167
POLAR_AC _21	47.43193	33.25267	12.5	1172.295	527.2636	68.94937	31.01138	2.223357	1.789738
POLAR_AC _22	59.38034	46.95091	26.24585	999.065	603.3861	62.28717	37.61839	1.655764	1.822622
POLAR_AC _23	45.14488	22.64495	3.404255	198.2039	183.1805	51.91873	47.9834	1.082014	1.794281
POLAR_AC _24	66.56113	43.76991	0.25641	619.7552	2333.334	20.98563	79.00939	0.265609	0.830911
POLAR_AC _25	32.67892	14.38971	0.588235	242.8614	54.31957	81.68547	18.27017	4.470974	1.701179

AC: R-R intervals imported from Polar V800™ and corrected by automatic correction method of Kubios HRV Premium (ver.3.2).

Raw HRV measures calculated from Kubios HRV Premium (ver.3.2) TBC Polar V800^{TM} R-R Intervals (mean±SD).

Subjects	SDNN (ms)	RMSSD (ms)	pNN50 (%)	LFpow_ (ms2)	HFpow_ (ms2)	LFpow_(n.u.)	HFpow_(n.u.)	LF_HF_ratio	SampEn
POLAR_TBC _1	93.19109	69.99802	44.68865	1912.907	1776.386	51.82974	48.13074	1.076853	1.606964
POLAR_TBC_2	66.43713	54.40181	24.31193	1788.846	593.0971	75.01797	24.87243	3.016109	1.485962
POLAR_TBC_3	75.93278	67.53018	42.44373	1620.694	1650.976	49.5168	50.442	0.981658	1.937668
POLAR_TBC_4	60.84045	28.10463	7.258065	2987.663	157.8171	94.97181	5.016689	18.93117	1.1418
POLAR_TBC_5	106.2346	49.63892	22.33677	1951.934	824.6232	70.26948	29.68637	2.367062	1.30353
POLAR_TBC _6	26.64506	13.12733	0	195.6638	78.70097	71.29155	28.67528	2.486168	1.758126
POLAR_TBC _7	82.41453	22.3191	1.466276	969.1428	114.719	89.38738	10.58093	8.447968	1.228302
POLAR_TBC _8	19.93159	13.32183	0.561798	130.0702	74.46039	63.55993	36.38573	1.746837	1.516484
POLAR_TBC _9	30.05709	16.11602	0.724638	376.7845	87.80853	81.08743	18.89719	4.290979	1.52678
POLAR_TBC _10	128.1552	127.2658	60.58824	6484.533	5965.021	52.06034	47.88949	1.087093	1.65726
POLAR_TBC _11	66.7831	37.749	16.80217	1551.825	350.1889	81.51344	18.39453	4.431394	1.744692
POLAR_TBC _12	55.00223	46.15264	11.5903	1171.035	761.3897	60.52385	39.3517	1.538024	1.662016
POLAR_TBC _13	63.35214	87.11615	25.88997	778.8463	1114.05	41.01313	58.66457	0.699112	0.853671
POLAR_TBC _14	32.67964	25.46359	3.75	162.4191	321.0508	33.56543	66.34815	0.505898	1.657119
POLAR_TBC _15	43.60694	20.58187	1.498929	1000.913	137.5481	87.86049	12.07402	7.276824	1.177978
POLAR_TBC _16	23.33778	7.933189	0	125.7425	35.71065	77.86363	22.11314	3.521147	1.323371
POLAR_TBC _17	74.90379	35.14285	11.64179	2293.492	374.9531	85.92485	14.04748	6.116744	1.400808
POLAR_TBC _18	49.03554	40.1668	14.66667	395.6216	1144.698	25.66485	74.25911	0.345612	1.586692
POLAR_TBC _19	67.5676	34.01589	11.16751	1327.143	458.1968	74.27682	25.6441	2.896449	1.528219
POLAR_TBC _20	20.11681	9.315384	0.246305	194.1833	34.9089	84.74011	15.23398	5.562573	1.462808
POLAR_TBC _21	47.8053	32.78967	11.36364	1112.984	585.4238	65.51387	34.45996	1.901159	1.630596
POLAR_TBC _22	59.38034	46.95091	26.24585	999.065	603.3861	62.28717	37.61839	1.655764	1.853573
POLAR_TBC _23	45.145	22.64495	3.404255	198.2039	183.1805	51.91873	47.9834	1.082014	1.763296
POLAR_TBC _24	30.448	22.16062	1.295337	236.0212	96.51789	70.85476	28.97516	2.445362	1.387696
POLAR_TBC _25	32.626	14.11557	0	263.1249	68.27335	79.36906	20.59399	3.853991	1.856722

TBC: R-R intervals imported from Polar V800^{TM} and corrected by threshold-based correction method of Kubios HRV Premium (ver.3.2).

Raw HRV measures calculated from MC V800^{TM} R-R Intervals (mean±SD).

Subjects	SDNN (ms)	RMSSD (ms)	pNN50 (%)	LFpow_ (ms2)	HFpow_ (ms2)	LFpow_(n.u.)	HFpow_(n.u.)	LF_HF_ratio	SampEn
POLAR_MC _1	92.24635	69.92936	44.48529	1912.907	1776.386	51.82974	48.13074	1.076853	1.586258
POLAR_MC_2	66.49921	54.44327	24.36782	1787.756	592.9379	75.01156	24.87879	3.015081	1.498238
POLAR_MC_3	86.8365	90.70041	42.58065	2235.976	2863.393	43.82027	56.1163	0.780883	1.691666
POLAR_MC_4	60.75149	28.17091	7.277628	2868.906	145.6132	95.15918	4.829867	19.70224	1.1418
POLAR_MC_5	82.46625	44.16878	20.48611	1575.129	474.6213	76.81712	23.1467	3.318707	1.591653
POLAR_MC _6	26.64506	13.12733	0	195.6638	78.70097	71.29155	28.67528	2.486168	1.758126
POLAR_MC _7	82.41453	22.3191	1.466276	969.1428	114.719	89.38738	10.58093	8.447968	1.228302
POLAR_MC _8	19.46523	11.03234	0	132.2477	72.71691	64.50697	35.46942	1.818665	1.504521
POLAR_MC _9	30.05709	16.11602	0.724638	376.7845	87.80853	81.08743	18.89719	4.290979	1.52678
POLAR_MC _10	128.1552	127.2658	60.58824	6484.533	5965.021	52.06034	47.88949	1.087093	1.60392
POLAR_MC _11	66.55504	37.78513	16.84783	1548.044	348.4661	81.55396	18.35787	4.442452	1.75275
POLAR_MC _12	54.78717	47.54851	11.22715	1046.683	782.2129	57.1437	42.70493	1.338105	1.515558
POLAR_MC _13	62.78986	86.23683	24.84076	768.0052	1064.148	41.7745	57.88273	0.721709	0.810567
POLAR_MC _14	32.67964	25.46359	3.75	162.4191	321.0508	33.56543	66.34815	0.505898	1.657119
POLAR_MC _15	43.65332	20.58099	1.502146	1000.913	137.5481	87.86049	12.07402	7.276824	1.136733
POLAR_MC _16	23.31523	7.942927	0	123.7421	36.02568	77.43297	22.54347	3.43483	1.323371
POLAR_MC _17	73.09747	34.15964	12.3494	2167.421	274.9371	88.72261	11.25445	7.883335	1.423671
POLAR_MC _18	48.40465	39.99263	14.4385	393.5114	1147.63	25.51428	74.40943	0.34289	1.523997
POLAR_MC _19	68.02752	34.90348	12.01117	1392.939	456.4016	75.26078	24.65947	3.052004	1.528219
POLAR_MC _20	20.00544	9.279213	0.239234	193.6813	35.57349	84.45566	15.51199	5.444541	1.44835
POLAR_MC _21	47.89232	33.57325	12.78196	1200.261	568.3553	67.84397	32.1259	2.111815	1.573437
POLAR_MC _22	59.45369	47.02327	26.33333	999.065	603.3861	62.28717	37.61839	1.655764	1.837526
POLAR_MC _23	45.14488	22.64495	3.404255	198.2039	183.1805	51.91873	47.9834	1.082014	1.763296
POLAR_MC _24	28.66669	14.05671	0.507614	222.1538	82.12563	72.96172	26.97243	2.705048	1.548677
POLAR_MC _25	32.60625	14.54988	0.588235	247.9823	58.37412	80.90855	19.04557	4.248155	1.83753

MC: R-R intervals imported from Polar V800^{TM} and corrected by manual correction method.
