# Supplementary material for: Validation of the Polar V800 heart rate monitor and comparison of artifact correction methods among adults with hypertension
Source: PLoS One. 2020 Oct 8;15(10):e0240220. doi: 10.1371/journal.pone.0240220 (PMC7544136; doi:10.1371/journal.pone.0240220)
Supplement: S1 Table — (PDF) [file pone.0240220.s008.pdf]

MEDICATION USE

S1A. Comparison of HRV measures separated by medication use calculated from UN Polar V800™ and ECG R-R intervals (mean ± SD)

HRV Measure	ECG (mean±SD)	Polar UN (mean±SD)	Bias (LoA)	ICC (95% CI)	Effect Size
**SDNN (ms)**					
Medication (n:6)	48.3±20.1	109.5±45.7	-61.24 (-143.20 to 20.71)	0.22 (-0.33-0.80)	1.734
Non-medication (n:19)	57.8±28.6	84.5±66.4	-26.64 (-139.35 to 80.07)	0.50 (-0.16-0.79)	0.522
**RMSSD (ms)**					
Medication (n:6)	36.2+30.1	133.2±79.3	-97.01 (-228.39 to 34.36)	0.29 (-0.33-0.83)	1.618
Non-medication (n:19)	39.0±29.9	80.1±99.6	-41.09 (-215.38 to 133.19)	0.38 (-0.38-0.75)	0.559
**pNN50 (%)**					
Medication (n:6)	8.8±10.4	11.8±11.0	-3.02 (-7.73 to 1.69)	0.97 (0.42-0.99)	0.280
Non-medication (n:19)	15.1±17.5	16.1±17.3	-0.98 (-5.32 to 3.37)	0.99 (0.98-0.99)	0.057
**LF (ms^2)**					
Medication (n:6)	920.1±751.9	6081.1±4949.6	-5161.09 (-15135.50 to 1413.31)	-0.36 (-0.89-0.75)	0.376
Non-medication (n:19)	1308.2±1497.5	7190.4±19386.3	-5882.20 (-43414.70 to 31650.32)	0.56 (-1.30-0.62)	0.347
**HF (ms^2)**					
Medication (n:6)	413.9±465.6	8171.5±6549.2	-7757.60 (-20069.90 to 4554.70	0.73 (-0.52-0.75)	1.671
Non-medication (n:19)	844.0±1420.9	3815.5±6789.5	-2971.52 (-15679.50 to 9736.5)	0.20 (-0.74-0.66)	0.606
**LF (nu)**					
Medication (n:6)	72.9±20.3	49.4±19.4	23.73 (-24.92 to 72.37)	0.24 (-0.73-0.85)	1.836

Non-medication (n:19)	65.5±18.3	63.1±19.8	2.42 (-25.7 to 30.6)	0.83 (0.58-0.93)	0.126
**HF (nu)**					
Medication (n:6)	27.0±20.2	50.7±19.4	-23.66 (-72.06 to 24.74)	0.24 (-0.73-0.85)	1.197
Non-medication (n:19)	34.5±18.3	36.9±19.8	-2.40 (-30.50 to 25.69)	0.83 (0.58-0.93)	0.126
**LF/HF Ratio**					
Medication (n:6)	4.5±3.0	1.8±2.7	2.64 (-3.49 to 8.76)	0.46 (-0.68-0.91)	0.946
Non-medication (n:19)	3.4±4.4	3.3±4.6	0.05 (-2.15 to 2.26)	0.98 (0.96-0.99)	0.022
**Sample Entropy**					
Medication (n:6)	1.4+0.3	0.7±0.3	0.70 (-0.12 to 1.51)	0.01 (-0.30-0.64)	2.332
Non-medication (n:19)	0.5+0.2	1.3±0.5	0.24 (-0.61 to 1.09)	0.30 (-0.45-0.70)	2.278

S1B. Comparison of HRV measures separated by medication use calculated from Kubios Premium (ver. 3.2) AC Polar V800™ and ECG R-R intervals (mean ± SD)

HRV Measure	ECG (mean±SD)	Polar AC (mean±SD)	Bias (LoA)	ICC (95% CI)	Effect Size
**SDNN (ms)**					
Medication (n:6)	48.3±20.1	46.9±25.1	1.34 (-25.36 to 28.04)	0.91 (0.34-0.98)	0.062
Non-medication (n:19)	57.8±28.6	60.3±28.9	-2.53 (-22.86 to 17.79)	0.96 (0.91-0.98)	0.087
**RMSSD (ms)**					
Medication (n:6)	36.2+30.1	25.8±12.9	10.44 (-35.82 to 56.70)	0.64 (-1.06-0.94)	0.449
Non-medication (n:19)	39.0±29.9	39.5±27.7	-0.49 (-18.18 to 17.20)	0.97 (0.93-0.99)	0.017
**pNN50 (%)**					
Medication (n:6)	8.8±10.4	5.8±5.8	2.99 (-12.88 to 18.80)	0.71 (-0.87-0.95)	0.356
Non-medication (n:19)	15.1±17.5	15.4±17.7	-0.28 (-1.27 to 0.71)	1.00 (0.99-1.00)	0.017
**LF (ms^2)**					
Medication (n:6)	920.1±751.9	917.4±910.0	2.62 (-452.35 to 457.58)	0.98 (0.88-0.99)	0.003
Non-medication (n:19)	1308.2±1497.5	1307.9±1483.5	0.23 (-353 to 354.18)	0.99 (0.99-0.99)	0.001
**HF (ms^2)**					
Medication (n:6)	413.9±465.6	255.3±225.8	158.5 (-659.04 to 976.11)	0.52 (-2.18-0.93)	0.433
Non-medication (n:19)	844.0±1420.9	905.0±1388.4	-60.95 (-1245.45 to 1123.554)	0.95 (0.88-0.98)	0.043
**LF (nu)**					
Medication (n:6)	72.9±20.3	78.3±10.6	-5.45 (-33.04 to 22.15)	0.77 (-0.42-0.96)	0.333
Non-medication (n:19)	65.5±18.3	63.0±20.6	2.47 (-21.02 to 25.97)	0.89 (0.73-0.96)	0.128

**HF (nu)**					
Medication (n:6)	27.0±20.2	21.6±10.6	5.41 (-22.04 to 32.86)	0.77 (-0.42-0.96)	0.336
Non-medication (n:19)	34.5±18.3	36.9±20.6	-2.48 (-25.99 to 21.04)	0.89 (0.73-0.96)	0.123
**LF/HF Ratio**					
Medication (n:6)	4.5±3.0	4.4±2.1	0.00 (-3.47 to 3.48)	0.99 (0.99-1.00)	0.039
Non-medication (n:19)	3.4±4.4	3.2±4.3	0.16 (-1.00 to 1.32)	0.99 (0.98-0.99)	0.046
**Sample Entropy**					
Medication (n:6)	1.4+0.3	1.4±0.2	-0.01 (-0.27 to 0.25)	0.94 (0.55-0.99)	0.038
Non-medication (n:19)	1.5+0.2	1.5±0.3	0.04 (-0.37 to 0.46)	0.72 (0.28-0.89)	0.177

S1C. Comparison of HRV measures separated by medication use calculated from Kubios Premium (ver. 3.2) TBC Polar V800^{TM} and ECG R-R intervals (mean ± SD)

HRV Measure	ECG (mean±SD)	Polar TBC (mean±SD)	Bias (LoA)	ICC (95% CI)	Effect Size
**SDNN (ms)**					
Medication (n:6)	48.3±20.1	47.3±20.2	-0.02 (-1.73 to 1.69)	1.00 (0.99-1.00)	0.050
Non-medication (n:19)	57.8±28.6	58.5±29.6	-0.72 (-12.3 to 10.82)	0.99 (0.97-0.99)	0.024
**RMSSD (ms)**					
Medication (n:6)	36.2±30.1	35.4±28.8	0.81 (-2.65 to 4.27)	0.99 (0.99-1.00)	0.027
Non-medication (n:19)	39.0±29.9	38.5±27.9	0.45 (-11.07 to 11.96)	0.99 (0.97-0.99)	0.017
**pNN50 (%)**					
Medication (n:6)	8.8±10.4	8.5±10.1	0.31 (-0.65 to 1.27)	0.99 (0.99-1.00)	0.029
Non-medication (n:19)	15.1±17.5	15.4±17.6	-0.31 (-1.41 to 0.80)	1.00 (0.99-1.00)	0.017
**LF (ms^2)**					
Medication (n:6)	920.1±751.9	950.3±765.3	-30.21 (-127.22 to 66.80)	0.99 (0.99-1.00)	0.040
Non-medication (n:19)	1308.2±1497.5	1290.9±1499.0	17.25 (-300.58 to 335.09)	0.99 (0.99-0.99)	0.012
**HF (ms^2)**					
Medication (n:6)	413.9±465.6	415.2±436.3	-1.31 (-105.09 to 102.5)	0.99 (0.98-1.00)	0.003
Non-medication (n:19)	844.0±1420.9	794.8±1354.9	49.19 (-520.7 to 619.04)	0.98 (0.97-0.99)	0.035
**LF (nu)**					
Medication (n:6)	72.9±20.3	73.2±18.7	-0.37 (-4.83 to 4.08)	0.99 (0.98-1.00)	0.015
Non-medication (n:19)	65.5±18.3	65.4±17.9	0.09 (-3.89 to 4.08)	0.99 (0.99-0.99)	0.006

**HF (nu)**					
Medication (n:6)	27.0±20.2	26.7±18.6	0.36 (-4.07 to 4.80)	0.99 (0.98-1.00)	0.015
Non-medication (n:19)	34.5±18.3	34.6±17.9	-0.09 (-4.05 to 3.88)	0.99 (0.99-0.99)	0.006
**LF/HF Ratio**					
Medication (n:6)	4.5±3.0	4.2±2.6	0.27 (-1.40 to 1.94)	0.99 (0.99-1.00)	0.107
Non-medication (n:19)	3.4±4.4	3.3±4.2	0.07 (-0.46 to 0.59)	0.99 (0.99-1.00)	0.023
**Sample Entropy**					
Medication (n:6)	1.4+0.3	1.4±0.4	-0.03 (-0.21 to 0.16)	0.98 (0.87-0.99)	0.063
Non-medication (n:19)	1.5+0.2	1.54±0.2	0.00 (-0.21 to 0.22)	0.91 (0.76-0.96)	0.052

S1D. Comparison of HRV measures separated by medication use calculated from MC Polar V800™ and ECG R-R intervals (mean ± SD)

HRV Measure	ECG (mean±SD)	Polar MC (mean±SD)	Bias (LoA)	ICC (95% CI)	Effect Size
**SDNN (ms)**					
Medication (n:6)	48.3±20.1	47.8±19.7	0.42 (-0.97 to 1.82)	1.00 (0.99-1.00)	0.025
Non-medication (n:19)	57.8±28.6	57.7±28.6	0.14 (-0.55 to 0.84)	1.00 (1.00-1.00)	0.003
**RMSSD (ms)**					
Medication (n:6)	36.2±30.1	35.4±28.5	0.82 (-2.6 to 4.2)	0.99 (0.99-1.00)	0.027
Non-medication (n:19)	39.0±29.9	38.9±30.0	-0.02 (-1.2 to 1.6)	1.00 (1.00-1.00)	0.003
**pNN50 (%)**					
Medication (n:6)	8.8±10.4	8.8±9.7	0.33 (-1.7 to 2.4)	0.97 (0.98-1.00)	0.009
Non-medication (n:19)	15.1±17.5	15.4±17.6	-0.25 (-1.3 to 0.8)	1.00 (0.99-1.00)	0.017
**LF (ms^2)**					
Medication (n:6)	920.1±751.9	904.1±718.2	3.98 (-68.70 to 76.7)	0.99 (0.99-1.00)	0.022
Non-medication (n:19)	1308.2±1497.5	1304.2±1499.9	15.94 (-60.2 to 92.1)	1.00 (1.00-1.00)	0.003
**HF (ms^2)**					
Medication (n:6)	413.9±465.6	392.1±429.1	21.75 (-67.13 to 110.63)	0.99 (0.98-1.00)	0.049
Non-medication (n:19)	844.0±1420.9	837.8±1428.1	6.21 (-32.2 to 44.7)	1.00 (1.00-1.00)	0.004
**LF (nu)**					
Medication (n:6)	72.9±20.3	73.5±19.4	-0.61 (-2.63 to 1.41)	0.99 (0.99-1.00)	0.030
Non-medication (n:19)	65.5±18.3	65.8±18.5	-0.27 (-1.32 to 0.79)	1.00 (0.99-1.00)	0.016

**HF (nu)**					
Medication (n:6)	27.0±20.2	26.4±19.3	0.61 (-1.42 to 2.65)	0.99 (0.99-1.00)	0.030
Non-medication (n:19)	34.5±18.3	34.2±18.5	0.27 (-0.79 to 1.32)	1.00 (0.99-1.00)	0.016
**LF/HF Ratio**					
Medication (n:6)	4.5±3.0	4.5±3.0	-0.04 (-0.32 to 0.24)	0.99 (0.99-1.00)	0.033
Non-medication (n:19)	3.4±4.4	3.4±4.4	-0.05 (-0.20 to 0.11)	1.00 (1.00-1.00)	0.033
**Sample Entropy**					
Medication (n:6)	1.4±0.3	1.4±0.4	0.02 (-0.14 to 0.17)	0.98 (0.91-0.99)	0.063
Non-medication (n:19)	1.5±0.2	1.5±0.2	0.00 (-0.10 to 0.10)	0.97 (0.94-0.99)	0.059
