# Supplementary material for: Validation of the Polar V800 heart rate monitor and comparison of artifact correction methods among adults with hypertension
Source: PLoS One. 2020 Oct 8;15(10):e0240220. doi: 10.1371/journal.pone.0240220 (PMC7544136; doi:10.1371/journal.pone.0240220)
Supplement: S2 Table — (PDF) [file pone.0240220.s009.pdf]

GENDER

S2A. Comparison of HRV measures separated by gender calculated from UN Polar V800^{TM} and ECG R-R intervals (mean ± SD)

HRV Measure	ECG (mean±SD)	Polar UN (mean±SD)	Bias (LoA)	ICC (95% CI)	Effect Size
**SDNN (ms)**					
Female (n:8)	35.4±13.5	78.2±67.0	-42.75 (-159.66 to 74.16)	0.31 (-0.86-0.83)	0.885
Male (n:17)	65.0±26.4	96.2±61.0	-31.27 (-138.74 to 76.19)	0.42 (-0.31-0.77)	0.665
**RMSSD (ms)**					
Female (n:8)	24.1±15.3	92.9±101.5	-68.75 (-255.35 to 117.84)	0.18 (-1.03-0.79)	0.948
Male (n:17)	45.0±32.4	92.8±97.0	-47.81 (-212.69 to 117.06)	0.43 (-0.30-0.77)	0.661
**pNN50 (%)**					
Female (n:8)	5.2±6.2	7.7±7.6	-2.49 (-9.08 to 4.10)	0.91 (0.51-0.98)	0.360
Male (n:17)	17.6±18.0	18.5±17.7	-0.98 (-4.27 to 2.30)	0.99 (0.99-0.99)	0.055
**LF (ms^2)**					
Female (n:8)	439.6±445.5	5199.1±6944.4	0.76 (-4759.46 to 6610.13)	0.13 (-1.12-0.78)	0.967
Male (n:17)	1579.9±1487.8	7736.0±20187.3	-6156.04 (-45404.60 to 33092.55)	0.04 (-1.49-0.64)	0.430
**HF (ms^2)**					
Female (n:8)	392.9±414.3	6571.6±8863.6	-6178.73 (-23007.90 to 10650.47)	0.03 (-1.28-0.75)	1.005
Male (n:17)	904.5±1492.6	4056.0±5960.6	-3151.45 (-14025.90 to 7722.97)	0.26 (-0.57-0.70)	0.725
**LF (nu)**					
Female (n:8)	58.4±18.9	50.5±18.3	7.91 (-14.67 to 30.49)	0.86 (0.33-0.97)	0.425

Male (n:17)	71.4±17.6	64.1±20.1	7.36 (-36.28 to 50.99)	0.45 (-0.40-0.79)	0.389
**HF (nu)**					
Female (n:8)	41.5±18.8	49.4±18.3	-7.87 (-30.35 to 14.61)	0.86 (0.33-0.97)	0.423
Male (n:17)	28.5±17.5	35.9±20.1	-7.33 (-50.85 to 36.18)	0.46 (-0.39-0.79)	0.388
**LF/HF Ratio**					
Female (n:8)	1.8±1.1	1.4±1.1	0.45 (-0.92 to 1.82)	0.85 (0.33-0.97)	0.412
Male (n:17)	4.5±4.6	3.7±4.9	0.78 (-4.07 to 5.63)	0.92 (0.79-0.97)	0.163
**Sample Entropy**					
Female (n:8)	1.5±0.3	1.0±0.6	0.42 (-0.60 to 1.45)	0.43 (-0.56-0.86)	0.891
Male (n:17)	1.6±0.2	1.3±0.5	0.28 (-0.58 to 1.15)	0.31 (-0.44-0.71)	0.821

S2B. Comparison of HRV measures separated by gender use calculated from Kubios Premium (ver. 3.2) AC Polar V800^{TM} and ECG R-R intervals (mean ± SD)

HRV Measure	ECG (mean±SD)	Polar AC (mean±SD)	Bias (LoA)	ICC (95% CI)	Effect Size
**SDNN (ms)**					
Female (n:8)	35.4±13.5	39.2±16.2	-3.77 (-31.35 to 23.82)	0.72 (-0.37-0.94)	0.253
Male (n:17)	65.0±26.4	65.6±28.9	-0.58 (-24.87 to 33.12)	0.97 (0.91-0.98)	0.021
**RMSSD (ms)**					
Female (n:8)	24.1±15.3	26.2±14.0	-2.44 (-26.49 to 22.27)	0.79 (-0.08-0.95)	0.145
Male (n:17)	45.0±32.4	40.8±28.5	4.13 (-24.87 to 33.12)	0.93 (0.82-0.97)	0.135
**pNN50 (%)**					
Female (n:8)	5.2±6.2	5.3±6.4	-0.14 (-1.20 to 0.93)	0.99 (0.99-1.00)	0.022
Male (n:17)	17.6±18.0	16.8±18.0	0.81 (-8.63 to 10.25)	0.98 (0.95-0.99)	0.045
**LF (ms^2)**					
Female (n:8)	439.6±445.47	441.8±375.4	-2.15 (-383.95 to 379.65)	0.94 (0.73-0.99)	0.005
Male (n:17)	1579.9±1487.8	1577.7±1509.2	2.19 (-374.41 to 378.80)	0.99 (0.98-0.99)	0.001
**HF (ms^2)**					
Female (n:8)	392.9±414.25	626.6±779.8	-233.73 (-1843.23 to 1375.77)	0.24 (-3.22-0.85)	0.374
Male (n:17)	904.5±1492.6	806.69±1423.96	97.83 (-652.59 to 848.25)	0.98 (0.95-0.99)	0.067
**LF (nu)**					
Female (n:8)	58.4±18.9	52.7±22.4	5.71 (-30.43 to 41.86)	0.75 (-0.14-0.95)	0.276
Male (n:17)	71.4±17.6	73.3±14.7	-1.85 (-18.61 to 14.92)	0.92 (0.79-0.97)	0.114

**HF (nu)**					
Female (n:8)	41.5±18.8	47.2±22.4	-5.72 (-41.91 to 30.46)	0.75 (-0.14-0.95)	0.276
Male (n:17)	28.5±17.5	26.7±14.6	1.84 (-14.85 to 18.52)	0.92 (0.80-0.97)	0.114
**LF/HF Ratio**					
Female (n:8)	1.8±1.1	1.6±1.2	0.24 (-1.44 to 1.91)	0.83 (0.22-0.96)	0.141
Male (n:17)	4.5±4.6	4.4±4.3	0.07 (-1.94 to 2.08)	0.98 (0.96-0.99)	0.022
**Sample Entropy**					
Female (n:8)	1.5±0.3	1.43±0.4	0.03 (-0.55 to 0.60)	0.79 (-0.14-0.96)	0.089
Male (n:17)	1.5±0.2	1.53±0.2	0.02 (-0.24 to 0.28)	0.87 (0.64-0.95)	0.052

S2C. Comparison of HRV measures separated by gender calculated from Kubios Premium (ver. 3.2) TBC Polar V800^{TM} and ECG R-R intervals (mean ± SD)

HRV Measure	ECG (mean±SD)	Polar TBC (mean±SD)	Bias (LoA)	ICC (95% CI)	Effect Size
**SDNN (ms)**					
Female (n:8)	35.4±13.5	35.6±13.2	-0.20 (-1.70 to 1.30)	0.99 (0.99-1.00)	0.015
Male (n:17)	65.0±26.4	65.7±27.6	-0.71 (-12.96 to 11.53)	0.98 (0.96-0.99)	0.026
**RMSSD (ms)**					
Female (n:8)	24.1±15.3	24.1±15.1	-1.02 (-7.8 to 5.8)	0.98 (0.93-0.99)	0.007
Male (n:17)	45.0±32.4	43.7±30.6	1.26 (-9.96 to 12.49)	0.99 (0.98-0.99)	0.041
**pNN50 (%)**					
Female (n:8)	5.2±6.2	5.4±6.1	-0.23 (-1.12 to 0.67)	0.99 (0.99-1.00)	0.033
Male (n:17)	17.6±18.0	17.7±18.0	-0.13 (-1.43 to 1.18)	1.00 (0.99-1.00)	0.006
**LF (ms^2)**					
Female (n:8)	439.6±445.5	441.2±441.2	-1.59 (-136.85 to 133.68)	0.99 (0.97-0.99)	0.004
Male (n:17)	1579.9±1487.8	1570.5±1491.7	9.37 (-323.64 to 342.37)	0.99 (0.99-0.99)	0.006
**HF (ms^2)**					
Female (n:8)	392.9±414.25	387.2±406.8	5.62 (-23.30 to 34.5)	1.00 (0.99-1.00)	0.014
Male (n:17)	904.5±1492.6	852.7±1421.2	51.87 (-555.04 to 658.77)	0.98 (0.97-0.99)	0.036
**LF (nu)**					
Female (n:8)	58.4±18.9	58.6±18.8	-0.17 (-3.28 to 2.95)	0.99 (0.99-1.00)	0.011
Male (n:17)	71.4±17.6	71.4±16.7	0.05 (-4.41 to 4.52)	0.99 (0.98-0.99)	0.006

**HF (nu)**					
Female (n:8)	41.5±18.8	41.3±18.8	0.18 (-2.89 to 3.25)	0.99 (0.99-1.00)	0.011
Male (n:17)	28.5±17.5	28.6±16.6	-0.06 (-4.51 to 4.40)	0.99 (0.98-0.99)	0.006
**LF/HF Ratio**					
Female (n:8)	1.8±1.1	1.8±1.1	0.00 (-0.23 to 0.23)	0.99 (0.98-0.99)	0.091
Male (n:17)	4.5±4.6	4.3±4.4	0.17 (-0.91 to 1.25)	0.99 (0.99-0.99)	0.044
**Sample Entropy**					
Female (n:8)	1.5±0.3	1.5±0.4	-0.02 (-0.21 to 0.18)	0.98 (0.90-0.99)	0.030
Male (n:17)	1.5±0.2	1.5±0.2	0.00 (-0.22 to 0.22)	0.90 (0.72-0.96)	0.054

S2D. Comparison of HRV measures separated by gender calculated from MC Polar V800™ and ECG R-R intervals (mean ± SD)

HRV Measure	ECG (mean±SD)	Polar MC (mean±SD)	Bias (LoA)	ICC (95% CI)	Effect Size
**SDNN (ms)**					
Female (n:8)	35.4±13.5	35.2±13.3	0.18 (-0.41 to 0.76)	1.00 (0.99-1.00)	0.015
Male (n:17)	65.0±26.4	64.8±26.3	0.23 (-0.82 to 1.27)	1.00 (1.00-1.00)	0.008
**RMSSD (ms)**					
Female (n:8)	24.1±15.3	24.1±14.9	0.03 (-1.00 to 1.06)	1.00 (0.99-1.00)	0.007
Male (n:17)	45.0±32.4	44.7±32.1	0.25 (-2.09 to 2.59)	1.00 (0.99-1.00)	0.009
**pNN50 (%)**					
Female (n:8)	5.2±6.2	5.3±6.4	-0.16 (-1.40 to 1.08)	0.99 (0.98-1.00)	0.016
Male (n:17)	17.6±18.0	17.7±17.9	-0.09 (-1.60 to 1.43)	1.00 (0.99-1.00)	0.006
**LF (ms^2)**					
Female (n:8)	439.6±445.5	434.6±435.4	5.03 (-25.01 to 35.06)	1.00 (0.99-1.00)	0.011
Male (n:17)	1579.9±1487.8	1572.2±1488.3	7.72 (-78.93 to 94.36)	1.00 (0.99-1.00)	0.005
**HF (ms^2)**					
Female (n:8)	392.9±414.3	368.1±410.9	6.76 (-25.11 to 38.63)	1.00 (0.99-1.00)	0.060
Male (n:17)	904.5±1492.6	893.1±1499.1	11.43 (-51.21 to 74.07)	1.00 (1.00-1.00)	0.008
**LF (nu)**					
Female (n:8)	58.4±18.9	58.8±19.1	-0.34 (-1.75 to 1.06)	1.00 (0.99-1.00)	0.021
Male (n:17)	71.4±17.6	71.8±17.4	-0.35 (-1.69 to 0.99)	1.00 (1.00-1.00)	0.023

**HF (nu)**					
Female (n:8)	41.5±18.8	41.2±19.1	0.35 (-1.05 to 1.75)	1.00 (0.99-1.00)	0.016
Male (n:17)	28.5±17.5	28.2±17.3	0.35 (-0.99 to 1.70)	1.00 (0.99-1.00)	0.017
**LF/HF Ratio**					
Female (n:8)	1.8±1.1	1.8±1.1	-0.03 (-0.20 to 0.13)	0.99 (0.99-1.00)	0.091
Male (n:17)	4.5±4.6	4.6±4.6	-0.05 (-0.24 to 0.14)	1.00 (0.99-1.00)	0.022
**Sample Entropy**					
Female (n:8)	1.5±0.3	1.5±0.4	-0.02 (-0.18 to 0.14)	0.98 (0.93-0.99)	0.030
Male (n:17)	1.5±0.2	1.5±0.2	0.02 (-0.07 to 0.11)	0.97 (0.93-0.99)	0.061
