# Supplementary material for: Validation of the Polar V800 heart rate monitor and comparison of artifact correction methods among adults with hypertension
Source: PLoS One. 2020 Oct 8;15(10):e0240220. doi: 10.1371/journal.pone.0240220 (PMC7544136; doi:10.1371/journal.pone.0240220)
Supplement: S3 Table — (PDF) [file pone.0240220.s010.pdf]

OBESITY

S3A. Comparison of HRV measures separated by BMI calculated from UN Polar V800^{TM} and ECG R-R intervals (mean ± SD)

HRV Measure	ECG (mean±SD)	Polar UN (mean±SD)	Bias (LoA)	ICC (95% CI)	Effect Size
**SDNN (ms)**					
Overweight (n:13)	59.9±31.1	96.2±73.1	-36.24 (-161.77 to 89.28)	0.46 (0.40-0.82)	0.645
Obese (n:12)	50.7±21.4	84.3±50.2	-33.54 (-125.96 to 58.89)	0.32 (-0.55-0.77)	0.869
**RMSSD (ms)**					
Overweight (n:13)	43.4±34.8	101.5±111.5	-58.05 (-252.89 to 136.80)	0.37 (-0.54-0.79)	0.703
Obese (n:12)	32.8±22.4	83.4±80.6	-50.69 (-195.59 to 94.22)	0.28 (-0.62-0.75)	0.856
**pNN50 (%)**					
Overweight (n:13)	17.0±20.1	18.6±19.5	-1.54 (-7.00 to 3.91)	0.99 (0.97-0.99)	0.078
Obese (n:12)	9.9±10.1	11.3±10.3	-1.38 (-5.27 to 2.51)	0.98 (0.93-0.99)	0.136
**LF (ms^2)**					
Overweight (n:13)	1694.1±1692.5	10043.26±23011.48	-8349.21 (-53196.80 to 36498.34)	0.03 (-1.83-0.69)	0.512
Obese (n:12)	696.1±546.7	3545.1±5001.7	-2849.05 (-12394.00 to 6695.86)	0.09 (-1.11-0.96)	0.801
**HF (ms^2)**					
Overweight (n:13)	1017.9±1693.6	5287.7±7379.0	-4269.84 (-18127.22 to 9587.54)	0.18 (-0.85-0.71)	0.780
Obese (n:12)	440.6±401.3	4398.7±6553.2	-3958.05 (-16605.20 to 8689.15)	0.05 (-1.13-0.67)	0.853
**LF (nu)**					
Overweight (n:13)	70.7±16.2	66.3±19.5	4.38 (-36.20 to 44.97)	0.51 (-0.65-0.85)	0.245

Obese (n:12)	63.5±21.1	52.6±19.4	10.94 (-23.75 to 45.64)	0.71 (0.08-0.91)	0.540
**HF (nu)**					
Overweight (n:13)	29.3±16.2	33.6±19.4	-4.36 (-44.86 to 36.15)	0.51 (-0.65-0.85)	0.244
Obese (n:12)	36.3±21.1	47.3±19.3	-10.92 (-45.44 to 23.61)	0.71 (0.09-0.91)	0.540
**LF/HF Ratio**					
Overweight (n:13)	4.4±5.1	4.0±5.3	0.42 (-4.35 to 5.18)	0.94 (0.82-0.98)	0.080
Obese (n:12)	2.8±2.3	1.9±2.3	0.95 (-2.24 to 4.15)	0.83 (0.40-0.95)	0.409
**Sample Entropy**					
Overweight (n:9)	1.5±0.2	1.2±0.5	0.30 (-0.66 to 1.25)	0.23 (-0.75-0.73)	0.789
Obese (n:12)	1.5±0.3	1.5±0.5	0.36 (-0.53 to 1.25)	0.49 (-0.30-0.83)	0.852

S3B. Comparison of HRV measures separated by BMI calculated from Kubios Premium (ver. 3.2) AC Polar V800^{TM} and ECG R-R intervals (mean ± SD)

HRV Measure	ECG (mean±SD)	Polar AC (mean±SD)	Bias (LoA)	ICC (95% CI)	Effect Size
**SDNN (ms)**					
Overweight (n:13)	59.9±31.1	59.4±30.4	0.57 (-5.66 to 6.81)	0.99 (0.99-0.99)	0.019
Obese (n:12)	50.7±21.4	50.7±24.9	0.06 (-18.20 to 18.32)	0.96 (0.86-0.98)	0.002
**RMSSD (ms)**					
Overweight (n:13)	43.4±34.8	43.4±31.6	-0.29 (-23.67 to 23.10)	0.97 (0.90-0.99)	0.004
Obese (n:12)	32.8±22.4	28.2±13.5	4.60 (-27.48 to 36.68)	0.75 (0.19-0.92)	0.249
**pNN50 (%)**					
Overweight (n:13)	17.0±20.1	17.2±20.2	-0.20 (-1.57 to 1.18)	1.00 (0.99-1.00)	0.010
Obese (n:12)	9.9±10.1	8.6±8.8	1.27 (-9.93 to 12.47)	0.90 (0.67-0.97)	0.249
**LF (ms^2)**					
Overweight (n:13)	1694.1±1692.5	1697.4±1698.9	-3.33 (-516.92 to 510.27)	0.99 (0.98-0.99)	0.002
Obese (n:12)	696.1±546.7	690.8±563.5	5.28 (-100.46 to 111.02)	0.99 (0.99-0.99)	0.134
**HF (ms^2)**					
Overweight (n:13)	1017.9±1693.6	1095.7±1641.7	-77.86 (-1538.99 to 1383.28)	0.95 (0.84-0.98)	0.047
Obese (n:12)	440.6±401.3	373.5±340.8	67.11 (-482.09 to 616.31)	0.83 (0.45-0.95)	0.010
**LF (nu)**					
Overweight (n:13)	70.7±16.2	67.1±20.2	3.57 (-24.99 to 32.14)	0.81 (0.41-0.94)	0.195
Obese (n:12)	63.5±21.1	66.2±19.9	-2.68 (-22.01 to 16.65)	0.93 (0.79-0.98)	0.180

**HF (nu)**					
Overweight (n:13)	29.3±16.2	32.8±20.2	-3.58 (-32.17 to 25.01)	0.81 (0.40-0.94)	0.196
Obese (n:12)	36.3±21.1	33.7±19.9	2.67 (-16.56 to 21.89)	0.94 (0.79-0.98)	0.131
**LF/HF Ratio**					
Overweight (n:13)	4.4±5.1	4.0±4.9	0.43 (-1.75 to 2.61)	0.98 (0.95-0.99)	0.085
Obese (n:12)	2.8±2.3	3.0±2.4	-0.21 (-1.50 to 1.08)	0.98 (0.93-0.99)	0.130
**Sample Entropy**					
Overweight (n:9)	1.5±0.2	1.5±0.3	0.05 (-0.36 to 0.47)	0.77 (0.28-0.93)	0.214
Obese (n:12)	1.5±0.3	1.5±0.3	-0.01 (-0.34 to 0.32)	0.98 (0.93-0.99)	0.088

S3C. Comparison of HRV measures separated by BMI calculated from Kubios Premium (ver. 3.2) TBC Polar V800™ and ECG R-R intervals (mean ± SD)

HRV Measure	ECG (mean±SD)	Polar TBC (mean±SD)	Bias (LoA)	ICC (95% CI)	Effect Size
**SDNN (ms)**					
Overweight (n:13)	59.9±31.1	59.4±30.4	0.54 (-5.71 to 6.79)	1.00 (1.00-1.00)	0.018
Obese (n:12)	50.7±21.4	52.5±25.1	-1.73 (-14.62 to 11.16)	0.98 (0.93-0.99)	0.074
**RMSSD (ms)**					
Overweight (n:13)	43.4±34.8	42.4±32.0	0.97 (-12.98 to 14.92)	1.00 (0.99-1.00)	0.029
Obese (n:12)	32.8±22.4	32.7±21.9	0.06 (-2.87 to 2.99)	0.99 (0.99-1.00)	0.003
**pNN50 (%)**					
Overweight (n:13)	17.0±20.1	17.2±20.1	-0.17 (-1.23 to 0.88)	1.00 (0.99-1.00)	0.008
Obese (n:12)	9.9±10.1	10.0±10.2	-0.14 (-1.48 to 1.20)	0.99 (0.99-1.00)	0.014
**LF (ms^2)**					
Overweight (n:13)	1694.1±1692.5	1673.1±1692.3	20.94 (-340.94 to 382.82)	1.00 (1.00-1.00)	0.012
Obese (n:12)	696.1±546.7	706.5±580.7	-10.48 (-178.27 to 157.32)	0.99 (0.98-0.99)	0.019
**HF (ms^2)**					
Overweight (n:13)	1017.9±1693.6	931.1±1619.1	86.74 (-580.93 to 754.41)	1.00 (0.99-1.00)	0.052
Obese (n:12)	440.6±401.3	457.4±403.9	-16.75 (-191.98 to 158.49)	0.98 (0.96-0.99)	0.042
**LF (nu)**					
Overweight (n:13)	70.7±16.2	71.2±14.9	-0.55 (-4.79 to 3.70)	1.00 (0.99-1.00)	0.035
Obese (n:12)	63.5±21.1	62.9±20.7	0.56 (-3.05 to 4.16)	0.99 (0.99-0.99)	0.027

**HF (nu)**					
Overweight (n:13)	29.3±16.2	28.7±14.9	0.55 (-3.66 to 4.77)	1.00 (0.99-1.00)	0.036
Obese (n:12)	36.3±21.1	36.9±20.7	-0.55 (-4.14 to 3.04)	0.99 (0.99-0.99)	0.027
**LF/HF Ratio**					
Overweight (n:13)	4.4±5.1	4.3±4.8	0.15 (-1.03 to 1.33)	0.99 (0.98-0.99)	0.031
Obese (n:12)	2.8±2.3	2.7±2.4	0.01 (-0.43 to 0.58)	0.99 (0.99-0.99)	0.032
**Sample Entropy**					
Overweight (n:13)	1.5±0.2	1.5±0.2	-0.02 (-0.20 to 0.17)	0.95 (0.81-0.98)	0.080
Obese (n:12)	1.5±0.3	1.5±0.3	0.01 (-0.22 to 0.25)	0.95 (0.83-0.98)	0.047

S3D. Comparison of HRV measures separated by BMI calculated from MC R-R intervals between the ECG and Polar (mean ± SD)

HRV Measure	ECG (mean±SD)	Polar MC (mean±SD)	Bias (LoA)	ICC (95% CI)	Effect Size
**SDNN (ms)**					
Overweight (n:13)	59.9±31.1	59.8±31.1	0.12 (-0.21 to 0.44)	1.00 (1.00-1.00)	0.004
Obese (n:12)	50.7±21.4	50.4±21.2	0.31 (-0.95 to 1.58)	1.00 (0.99-1.00)	0.015
**RMSSD (ms)**					
Overweight (n:13)	43.4±34.8	43.5±34.8	-0.05 (-1.01 to 0.9)	1.00 (1.00-1.00)	0.001
Obese (n:12)	32.8±22.4	32.3±21.4	0.43 (-2.27 to 3.12)	0.99 (0.99-1.00)	0.019
**pNN50 (%)**					
Overweight (n:13)	17.0±20.1	17.1±20.1	-0.10 (-1.15 to 0.95)	1.00 (1.00-1.00)	0.005
Obese (n:12)	9.9±10.1	10.0±9.9	-0.12 (-1.89 to 1.65)	0.99 (0.99-0.99)	0.012
**LF (ms^2)**					
Overweight (n:13)	1694.1±1692.5	1690.8±1693.4	3.29 (-60.69 to 67.26)	1.00 (1.00-1.00)	0.002
Obese (n:12)	696.1±546.7	685.3±829.8	10.72 (-72.49 to 93.92)	0.99 (0.99-1.00)	0.020
**HF (ms^2)**					
Overweight (n:13)	1017.9±1693.6	1015.9±1701.6	1.89 (-24.98 to 28.77)	1.00 (1.00-1.00)	0.001
Obese (n:12)	440.6±401.3	421.9±380.1	18.65 (-52.02 to 89.32)	0.99 (0.99-0.99)	0.048
**LF (nu)**					
Overweight (n:13)	70.7±16.2	71.00±16.1	-0.31 (-1.22 to 0.59)	1.00 (1.00-1.00)	0.019
Obese (n:12)	63.5±21.1	63.9±21.1	-0.39 (-2.11 to 1.34)	1.00 (0.99-1.00)	0.018
**HF (nu)**					
Overweight (n:13)	29.3±16.2	28.9±16.1	0.31 (-0.59 to 1.22)	1.00 (0.99-1.00)	0.020

Obese (n:12)	36.3±21.1	35.9±21.0	0.39 (-1.34 to 2.12)	1.00 (0.99-1.00)	0.018
**LF/HF Ratio**					
Overweight (n:13)	4.4±5.1	4.5±5.1	-0.04 (-0.23 to 0.16)	1.00 (1.00-1.00)	0.007
Obese (n:12)	2.8±2.3	2.9±2.4	-0.05 (-0.23 to 0.13)	1.00 (0.99-1.00)	0.022
**Sample Entropy**					
Overweight (n:13)	1.5±0.2	1.5±0.2	-0.01 (-0.20 to 0.17)	0.94 (0.82-0.98)	0.073
Obese (n:12)	1.5±0.3	1.5±0.3	0.01 (-0.14 to 0.15)	0.98 (0.94-0.99)	0.024
