# Supplementary material for: Validation of the Polar V800 heart rate monitor and comparison of artifact correction methods among adults with hypertension
Source: PLoS One. 2020 Oct 8;15(10):e0240220. doi: 10.1371/journal.pone.0240220 (PMC7544136; doi:10.1371/journal.pone.0240220)
Supplement: S4 Table — (PDF) [file pone.0240220.s011.pdf]

AEROBIC CAPACITY

S4A. Comparison of HRV measures separated by aerobic capacity calculated from UN Polar V800^{TM} and ECG R-R intervals (mean ± SD)

HRV Measure	ECG (mean±SD)	Polar UN (mean±SD)	Bias (LoA)	ICC (95% CI)	Effect Size
**SDNN (ms)**					
Very Poor to Poor (n:14)	47.9±19.1	78.3±50.9	-30.35 (-125.15 to 64.44)	0.28 (-0.59-0.73)	0.789
Fair to Good (n:8)	59.9±36.9	57.2±37.4	2.67 (-12.46 to 17.79)	0.58 (-0.57-0.91)	0.555
Excellent (n:3)	78.9±10.9	166.2±109.6	-87.21 (-283.70 to 109.28)	0.22 (-0.18-0.97)	1.120
**RMSSD (ms)**					
Very Poor to Poor (n:14)	26.4±11.6	75.9±81.3	-49.49 (-202.46 to 103.47)	0.13 (-0.84-0.67)	0.852
Fair to Good (n:8)	49.5±43.1	85.1±76.1	-35.57 (-168.73 to 97.59)	0.53 (-0.74-0.90)	0.575
Excellent (n:3)	63.6±23.6	192.1±176.3	-128.47 (-431.90 to 174.96)	0.32 (-0.51-0.97)	1.021
**pNN50 (%)**					
Very Poor to Poor (n:14)	6.2±6.1	8.1±7.1	-1.93 (-7.40 to 3.55)	0.93 (0.72-0.98)	0.291
Fair to Good (n:8)	20.6±22.6	21.8±22.3	-1.22 (-4.87 to 2.43)	0.99 (0.98-1.00)	0.054
Excellent (n:3)	29.5±12.2	29.5±11.6	0.04 (-1.07 to 1.14)	1.00 (0.98-1.00)	0.003
**LF (ms^2)**					
Very Poor to Poor (n:14)	782.8±661.9	3635.9±5467.7	-2853.04 (-13382.30 to 7676.26)	0.07 (-1.12-0.66)	0.733
Fair to Good (n:8)	1712.4±2138.1	3947.2±3923.7	-2234.83 (-10334.9 to 5865.19)	0.22 (-0.60-0.72)	0.707
Excellent (n:3)	1905.7±280.7	30208.0±47686.0	-28302.36 (-121227.00 to 64622.01)	0.02 (-0.12-0.97)	0.839

**HF (ms^2)**					
Very Poor to Poor (n:14)	335.5±321.2	4426.6±7090.6	-4091.12 (-17814.00 to 9631.77)	0.04 (-1.07-0.64)	0.815
Fair to Good (n:8)	1229.1±2004.9	4166.5±5424.7	-2937.43 (-13638.60 to 7763.72)	0.17 (-0.27-0.81)	0.718
Excellent (n:3)	1329.9±1331.1	8739.8±10476.3	-7409.76 (-25418.60 to 10599.12)	0.32 (-0.52-0.98)	0.992
**LF (nu)**					
Very Poor to Poor (n:14)	67.4±19.6	56.4±21.4	10.95 (-24.18 to 46.07)	0.71 (0.41-0.90)	0.532
Fair to Good (n:8)	67.9±19.4	63.3±19.3	4.63 (-25.79 to 35.05)	0.81 (0.12-0.96)	0.239
Excellent (n:3)	64.9±18.5	65.6±21.2	-0.65 (-70.91 to 69.60)	0.86 (0.69-0.92)	0.033
**HF (nu)**					
Very Poor to Poor (n:14)	32.6±19.6	43.5±21.4	-10.90 (-45.89 to 24.10)	0.71 (0.15-0.90)	0.531
Fair to Good (n:8)	32.0±19.3	36.7±19.3	-4.63 (-34.90 to 25.64)	0.81 (0.12-0.96)	0.240
Excellent (n:3)	34.9±18.5	34.3±21.2	0.66 (-69.56 to 70.88)	0.85 (0.69-0.92)	0.033
**LF/HF Ratio**					
Very Poor to Poor (n:14)	3.4±2.7	2.4±2.6	1.00 (-3.20 to 5.19)	0.78 (0.36-0.93)	0.373
Fair to Good (n:8)	4.7±6.3	4.2±6.7	0.47 (-2.89 to 3.83)	0.98 (0.92-0.99)	0.073
Excellent (n:3)	2.3±1.4	2.6±1.8	-0.29 (-6.06 to 5.48)	0.0.97 (0.94-0.99)	0.184
**Sample Entropy**					
Very Poor to Poor (n:14)	1.6±0.2	1.2±0.5	0.34 (-0.56 to 1.24)	0.32 (-0.45-0.74)	0.892
Fair to Good (n:8)	1.4±0.3	1.2±0.6	0.19 (-0.65 to 1.04)	0.70 (-0.25-0.93)	0.422
Excellent (n:3)	1.6±0.1	0.9±0.5	0.65 (-0.46 to 1.75)	-0.45 (-1.02-0.86)	1.951

S4B. Comparison of HRV measures separated by aerobic capacity calculated from Kubios Premium (ver. 3.2) AC Polar V800^{TM} and ECG R-R intervals (mean ± SD)

HRV Measure	ECG (mean±SD)	Polar AC (mean±SD)	Bias (LoA)	ICC (95% CI)	Effect Size
**SDNN (ms)**					
Very Poor to Poor (n:14)	47.9±19.1	51.6±21.4	-3.61 (-26.27 to 19.04)	0.91 (0.72-0.97)	0.178
Fair to Good (n:8)	59.9±36.9	57.2±37.4	2.67 (-12.46 to 17.79)	0.98 (0.95-0.99)	0.072
Excellent (n:3)	78.9±10.9	82.5±20.5	-3.60 (-36.54 to 29.34)	0.71 (0.59-0.99)	0.220
**RMSSD (ms)**					
Very Poor to Poor (n:14)	26.4±11.6	28.2±11.1	-1.77 (-20.10 to 16.56)	0.80 (0.38-0.93)	0.156
Fair to Good (n:8)	49.5±43.1	42.4±40.1	7.10 (-32.07 to 46.27)	0.93 (0.72-0.98)	0.171
Excellent (n:3)	63.6±23.6	59.5±10.0	7.09 (-19.90 to 34.09)	0.84 (-0.37-0.99)	0.392
**pNN50 (%)**					
Very Poor to Poor (n:14)	6.2±6.1	6.5±6.4	-0.28 (-1.64 to 1.07)	0.99 (0.98-0.99)	0.045
Fair to Good (n:8)	20.6±22.6	18.4±23.2	2.14 (-11.50 to 15.78)	0.97 (0.89-0.99)	0.094
Excellent (n:3)	29.5±12.2	29.7±11.4	-0.17 (-1.96 to 0.78)	0.99 (0.96-1.00)	0.015
**LF (ms^2)**					
Very Poor to Poor (n:14)	782.8±661.9	811.8±713.8	-28.95 (-380.13 to 322.22)	0.98 (0.95-0.99)	0.042
Fair to Good (n:8)	1712.4±2138.1	1718.7±2159.5	-6.34 (-1558.50 to 142.82)	1.00 (0.99-1.00)	0.003
Excellent (n:3)	1905.7±280.7	1746.9±113.7	158.72 (-614.13 to 931.56)	0.26 (0.37-0.87)	0.741
**HF (ms^2)**					
Very Poor to Poor (n:14)	335.5±321.2	448.1±605.9	-152.59 (-1357.04 to 1051.85)	0.33 (-1.07-0.78)	0.315

Fair to Good (n:8)	1229.1±2004.9	1113.7±2045.1	115.38 (-528.49 to 759.24)	0.99 (0.97-0.99)	0.057
Excellent (n:3)	1329.9±1331.1	994.5±560.3	335.49 (-1202.01 to 1873.00)	0.84 (-0.71-0.99)	0.329
**LF (nu)**					
Very Poor to Poor (n:14)	67.4±19.6	63.8±22.8	3.57 (-23.64 to 30.79)	0.88 (0.64-0.96)	0.168
Fair to Good (n:8)	67.9±19.4	72.3±15.7	-4.39 (-27.42 to 18.65)	0.87 (0.43-0.97)	0.248
Excellent (n:3)	64.9±18.5	65.2±13.5	-0.20 (-11.41 to 11.01)	0.97 (-0.41-0.99)	0.012
**HF (nu)**					
Very Poor to Poor (n:14)	32.6±19.6	36.1±22.8	-3.58 (-30.82 to 23.66)	0.88 (0.64-0.96)	0.168
Fair to Good (n:8)	32.0±19.3	27.7±15.7	4.36 (-18.55 to 27.28)	0.87 (0.43-0.97)	0.248
Excellent (n:3)	34.9±18.5	34.8±13.6	0.20 (-10.98 to 11.38)	0.97 (-0.39-0.99)	0.012
**LF/HF Ratio**					
Very Poor to Poor (n:14)	3.4±2.7	3.0±2.6	0.32 (-1.81 to 2.45)	0.95 (0.86-0.98)	0.123
Fair to Good (n:8)	4.7±6.3	4.9±5.9	-0.26 (-1.83 to 1.32)	0.99 (0.98-0.99)	0.042
Excellent (n:3)	2.3±1.4	2.1±1.0	0.19 (-0.82 to 1.20)	0.96 (0.04-0.99)	0.157
**Sample Entropy**					
Very Poor to Poor (n:14)	1.6±0.2	1.5±0.3	0.05 (-0.39 to 0.48)	0.74 (0.21-0.91)	0.186
Fair to Good (n:8)	1.4±0.3	1.5±0.2	-0.03 (-0.15 to 0.09)	0.98 (0.92-0.99)	0.115
Excellent (n:3)	1.6±0.1	1.6±0.3	0.02 (-0.50 to 0.55)	0.65 (0.51-0.99)	0.098

S4C. Comparison of HRV measures separated by aerobic capacity calculated from Kubios Premium (ver. 3.2) TBC Polar V800^{TM} and ECG R-R intervals (mean ± SD)

HRV Measure	ECG (mean±SD)	Polar TBC (mean±SD)	Bias (LoA)	ICC (95% CI)	Effect Size
**SDNN (ms)**					
Very Poor to Poor (n:14)	47.9±19.1	48.2±19.1	-0.21 (-1.54 to 1.12)	1.00 (0.99-1.00)	0.011
Fair to Good (n:8)	59.9±36.9	59.8±37.0	0.10 (-1.03 to 1.24)	1.00 (1.00-1.00)	0.008
Excellent (n:3)	78.9±10.9	82.9±20.8	-3.88 (-37.35 to 29.60)	0.71 (4.75-0.99)	0.036
**RMSSD (ms)**					
Very Poor to Poor (n:14)	26.4±11.6	27.1±10.6	-0.68 (-5.80 to 4.45)	0.98 (0.95-0.99)	0.061
Fair to Good (n:8)	49.5±43.1	49.1±42.8	0.44 (-1.95 to 2.82)	1.00 (0.99-1.00)	0.013
Excellent (n:3)	63.6±23.6	57.2±9.2	6.44 (-21.72 to 34.61)	0.82 (-5.02-0.99)	0.022
**pNN50 (%)**					
Very Poor to Poor (n:14)	6.2±6.1	6.4±6.1	-0.16 (-1.07 to 0.74)	0.99 (0.99-1.00)	0.027
Fair to Good (n:8)	20.6±22.6	20.7±22.8	-0.14 (-1.36 to 1.08)	1.00 (0.99-1.00)	0.001
Excellent (n:3)	29.5±12.2	29.7±11.1	-0.18 (-2.62 to 2.26)	0.99 (0.93-1.00)	0.031
**LF (ms^2)**					
Very Poor to Poor (n:14)	782.8±661.9	786.3±663.8	-3.46 (-104.34 to 97.42)	0.99 (0.99-1.00)	0.005
Fair to Good (n:8)	1712.4±2138.1	1732.5±2152.2	-20.07 (-146.77 to 106.63)	1.00 (0.99-1.00)	0.002
Excellent (n:3)	1905.65±280.68	1787.2±165.6	118.50 (-744.24 to 981. 24)	0.77 (0.46-0.78)	0.127
**HF (ms^2)**					
Very Poor to Poor (n:14)	335.5±321.2	339.2±314.9	-3.73 (-60.24 to 52.77)	0.99 (0.99-0.99)	0.012

Fair to Good (n:8)	1229.1±2004.9	1221.9±2015.4	7.21 (-51.04 to 65.45)	1.00 (1.00-1.00)	0.007
Excellent (n:3)	1329.9±1331.1	1022.9±556.1	307.10 (-1261.55 to 1875.75)	0.84 (-0.61-0.99)	0.015
**LF (nu)**					
Very Poor to Poor (n:14)	67.4±19.6	67.2±19.3	0.14 (-2.71 to 3.00)	0.99 (0.99-1.00)	0.007
Fair to Good (n:8)	67.9±19.4	68.2±19.1	-0.32 (-1.72 to 1.08)	1.00 (0.99-1.00)	0.018
Excellent (n:3)	64.9±18.5	64.9±13.6	0.04 (-11.45 to 11.53)	0.97 (-0.57-0.99)	0.013
**HF (nu)**					
Very Poor to Poor (n:14)	32.6±19.6	32.7±19.3	-0.14 (-2.97 to 2.70)	0.99 (0.99-1.00)	0.007
Fair to Good (n:8)	32.0±19.3	31.7±19.0	0.32 (-1.08 to 1.71)	1.00 (0.99-1.00)	0.018
Excellent (n:3)	34.9±18.5	35.0±13.6	-0.04 (-11.50 to 11.42)	0.97 (-0.55-0.99)	0.013
**LF/HF Ratio**					
Very Poor to Poor (n:14)	3.4±2.7	3.2±2.5	0.14 (-0.92 to 1.20)	0.98 (0.96-0.99)	0.052
Fair to Good (n:8)	4.7±6.3	4.6±6.1	0.04 (-0.53 to 0.62)	0.99 (0.99-1.00)	0.005
Excellent (n:3)	2.3±1.4	2.1±1.0	0.21 (-0.86 to 1.28)	0.95 (-0.06-0.99)	0.027
**Sample Entropy**					
Very Poor to Poor (n:14)	1.6±0.2	1.6±0.2	-0.01 (-0.18 to 0.17)	0.95 (0.84-0.98)	0.039
Fair to Good (n:8)	1.4±0.3	1.4±0.3	0.0 (-0.12 to 0.11)	0.99 (0.95-0.99)	0.052
Excellent (n:3)	1.6±0.1	1.6±0.3	0.64 (-0.50 to 0.55)	0.65 (0.55-0.99)	0.056

S4D. Comparison of HRV measures separated by aerobic capacity calculated from MC Polar V800^{TM} and ECG R-R intervals (mean ± SD)

HRV Measure	ECG (mean±SD)	Polar MC (mean±SD)	Bias (LoA)	ICC (95% CI)	Effect Size
**SDNN (ms)**					
Very Poor to Poor (n:14)	47.9±19.1	47.9±19.1	0.11 (-0.46 to 0.68)	1.00 (1.00-1.00)	0.006
Fair to Good (n:8)	59.9±36.9	59.6±36.9	0.31 (-0.92 to 1.55)	1.00 (1.00-1.00)	0.008
Excellent (n:3)	78.9±10.9	78.6±10.7	0.39 (-0.96 to 1.74)	0.99 (0.98-1.00)	0.036
**RMSSD (ms)**					
Very Poor to Poor (n:14)	26.4+11.6	26.6±11.5	-0.10 (-1.08 to 0.88)	1.00 (0.99-1.00)	0.009
Fair to Good (n:8)	49.5+43.1	49.0±42.7	0.54 (-2.45 to 3.53)	1.00 (0.99-1.00)	0.013
Excellent (n:3)	63.6+23.6	63.1±24.4	0.53 (-1.86 to 2.92)	0.99 (0.99-1.00)	0.022
**pNN50 (%)**					
Very Poor to Poor (n:14)	6.2±6.1	6.5±6.3	-0.28 (-1.42 to 0.86)	0.99 (0.99-0.99)	0.045
Fair to Good (n:8)	20.6±22.6	20.6±22.8	0.00 (-1.87 to 1.87)	1.00 (0.99-1.00)	0.001
Excellent (n:3)	29.5±12.2	29.1±11.8	0.37 (-0.49 to 1.24)	1.00 (0.99-1.00)	0.031
**LF (ms^2)**					
Very Poor to Poor (n:14)	782.8±661.9	776.9±647.3	5.83 (-59.04 to 70.71)	0.99 (0.99-1.00)	0.009
Fair to Good (n:8)	1712.4±2138.1	1715.9±2143.6	-3.56 (-50.45 to 43.33)	1.00 (0.99-1.00)	0.002
Excellent (n:3)	1905.7±280.7	1866.3±337.4	39.37 (-103.65 to 182.39	0.98 (0.78-1.00)	0.127
**HF (ms^2)**					
Very Poor to Poor (n:14)	335.5±321.2	330.5±318.5	5.05 (-18.85 to 28.94)	1.00 (0.99-1.00)	0.016

Fair to Good (n:8)	1229.1±2004.9	1214.3±2016.7	14.85 (-71.32 to 101.01)	1.00 (1.00-1.00)	0.007
Excellent (n:3)	1329.9±1331.1	1310.3±1346.3	19.68 (-42.87 to 82.23)	1.00 (0.99-1.00)	0.015
**LF (nu)**					
Very Poor to Poor (n:14)	67.4±19.6	67.8±19.8	-0.37 (-1.42 to 0.68)	1.00 (0.99-1.00)	0.019
Fair to Good (n:8)	67.9±19.4	68.3±18.9	-0.35 (-2.31 to 1.61)	1.00 (0.99-1.00)	0.018
Excellent (n:3)	64.9±18.5	65.2±18.6	-0.24 (-0.99 to 0.51)	1.00 (0.99-1.00)	0.013
**HF (nu)**					
Very Poor to Poor (n:14)	32.6±19.6	32.2±19.8	0.37 (-0.68-1.42)	1.00 (0.99-1.00)	1.784
Fair to Good (n:8)	32.0±19.3	31.7±18.8	0.35 (-1.62 to 2.32)	1.00 (0.99-1.00)	0.018
Excellent (n:3)	34.9±18.5	34.7±18.6	0.25 (-0.51 to 1.00)	1.00 (0.99-1.00)	0.013
**LF/HF Ratio**					
Very Poor to Poor (n:14)	3.4±2.7	3.4±2.7	-0.05 (-0.25 to 0.14)	1.00 (0.99-1.00)	0.020
Fair to Good (n:8)	4.7±6.3	4.7±6.3	-0.03 (-0.22 to 0.16)	0.99 (0.99-1.00)	0.005
Excellent (n:3)	2.3±1.4	2.4±1.4	-0.04 (-0.18 to 0.11)	0.99 (0.98-1.00)	0.027
**Sample Entropy**					
Very Poor to Poor (n:14)	1.6±0.2	1.6±0.2	0.00 (-0.13 to 0.13)	0.97 (0.91-0.99)	0.014
Fair to Good (n:8)	1.4±0.3	1.4±0.3	0.02 (-0.11 to 0.14)	0.99 (0.95-0.99)	0.052
Excellent (n:3)	1.6±0.1	1.6±0.1	0.01 (-0.03 to 0.04)	0.99 (0.87-1.00)	0.056
